# Urinary amino acid metabolomic profiling and its association with childhood obesity in prepubescent individuals

**DOI:** 10.3389/fphys.2025.1524939

**Published:** 2025-04-29

**Authors:** Mariana Doce Passadore, Nayara Azinheira Nobrega Cruz, Mariana Zuccherato Bocato, Leonardo de Abreu Ferreira, Marcelo Yudi Icimoto, Maria del Carmen Bisi Molina, José Geraldo Mill, Fernando Barbosa Junior, Dulce Elena Casarini, Lilian Caroline Gonçalves de Oliveira

**Affiliations:** ^1^ Postgraduation Program of Nephrology, Nephrology Division, Department of Medicine, Universidade Federal de São Paulo (UNIFESP/EPM), Sao Paulo, Brazil; ^2^ Postgraduation Program of Translational Medicine, Nephrology Division, Department of Medicine, Universidade Federal de São Paulo (UNIFESP/EPM), Sao Paulo, Brazil; ^3^ Analytical and System Toxicology Laboratory, Department of Clinical, Toxicological and Bromatological Analysis, Faculty of Pharmaceutical Sciences of Ribeirao Preto, Universidade de São Paulo, Sao Paulo, Brazil; ^4^ Universidade Presbiteriana Mackenzie, Sao Paulo, Brazil; ^5^ Department of Biophysics, Universidade Federal de São Paulo (UNIFESP/EPM), Sao Paulo, Brazil; ^6^ Department of Integrated Health Education, Center for Health Sciences, Universidade Federal do Espírito Santo, Vitória, Brazil; ^7^ Department of Physiological Sciences, Universidade Federal do Espírito Santo, Vitória, Brazil; ^8^ Nephrology Division, Department of Medicine, Universidade Federal de São Paulo (UNIFESP/EPM), Sao Paulo, Brazil

**Keywords:** childhood obesity, prepuberty, urinary amino acids, metabolomics, biomarkers

## Abstract

**Introduction:**

Amino acids are fundamental in several metabolic processes, and their levels can reflect metabolism impairments that contribute to obesity and related diseases. Our objective was to identify a urinary amino acid fingerprint in obese and overweight children in prepuberty and to correlate this profile with cardiometabolic alterations.

**Methods:**

The study included 110 children, boys and girls aged 9–10 years, they were classified according to their BMI-for-age (Body Mass Index for age) into three groups: normal weight (NW) (n = 45), overweight (OW) (n = 21), and obese (OB) (n = 44). The 12-h urine samples were analyzed by LC-MS/MS to quantify 47 amino acids using the *Amino Acids Analysis Kit* (Zivak®, Turkey), values were corrected by creatinine concentration. Anthropometric measurements, cardiovascular parameters, and biochemical profiles were assessed following standard protocols.

**Results:**

When compared to NW, anthropometric measures, systolic and diastolic blood pressure, and serum uric acid levels were progressively elevated in the OW and OB groups. The OB group was characterized by elevated alpha-aminoadipic acid, asparagine, cystathionine, 1-methyl-histidine, serine, tryptophan, phenylalanine, and tyrosine. In contrast, the OW group presented the most expressive levels of glutamine, alpha-diaminopimelic, and sarcosine.

**Discussion:**

Our findings indicate that obese and overweight children exhibit a particular urinary amino acid fingerprint which is similar to that reported in studies with plasma. The altered amino acids, particularly tyrosine, are frequently associated with impairments in glucose homeostasis, insulin resistance, and diabetes mellitus type 2. Potential mechanisms for increasing the levels of these amino acids in excess of weight may include enhanced protein degradation and impaired oxidative metabolism.

## Introduction

Childhood obesity prevalence has progressively increased worldwide. Over 340 million children and adolescents aged 5–19 years were overweight or obese in 2016 (World Health Organization, 2020; Singh et al., 2023). According to the World Obesity Federation’s 2019 estimates, 206 million children and adolescents aged 5 to 19 will be obese by 2025, which is expected to increase to 254 million by 2030. Excessive fat accumulation in childhood is associated with an increased risk of the early development of cardiovascular diseases (Stefan, 2020), insulin resistance, diabetes mellitus (Hameed et al., 2020), and cancers (Di Angelantonio et al., 2016). Discovering biomarkers of predisposition to obesity and related comorbidities enables early diagnosis, identifying individuals who better benefit from non-medical interventions such as physical activity and healthy diet, and who need early treatment, thus contributing to disease prevention or better prognostic (Dessì et al., 2014).

Metabolomics is an emerging field recognized as a powerful tool for discovering the metabolic fingerprint of a given phenotypic change (Su et al., 2014). The metabolome represents the total metabolites in a given organism, such as sugars, organic acids, amino acids, lipids, vitamins, minerals, and other compounds. Genetic and epigenetic factors, age, environment, nutrition, drugs, and lifestyle reflect individual metabolome alterations. Moreover, the metabolites are not inert molecules, they can directly or indirectly regulate gene and protein expression and participate in the maintenance of homeostasis (Gerszten and Wang, 2008; Scalabre et al., 2017). Several metabolites have been correlated to disease phenotypes, as their presence or concentrations in biological fluids are directly related to pathogenic mechanisms (Su et al., 2014; Scalabre et al., 2017). Therefore, metabolomics enables the identification of biomarkers of exposure or susceptibility to several diseases, providing remarkable opportunities for better understanding exposure and predicting potential adverse health outcomes (Dessì et al., 2014; Handakas et al., 2022).

Obesity is a complex phenotype depending on several genetic and lifestyle factors. Despite being extensively studied, there is still no complete elucidation of the mechanisms underlying its development, particularly in childhood. Recent metabolomic studies have identified that the concentration of several metabolites including amino acids, lipids, monosaccharides, organic acids, and serotonin are altered in patients with obesity, thus being potential candidates as biomarkers (Hameed et al., 2020; Zhang et al., 2013; Rangel-Huerta et al., 2019; Szczerbinski et al., 2022). Nonetheless, there are fewer metabolomic studies targeting childhood obesity, partly due to methodological and ethical challenges in obtaining blood samples. These studies also need to be expanded, considering the bases of obesity, growth, and pubertal hormones.

Human urine is a rich biofluid for metabolomic studies, since it concentrates a wide range of metabolites that change with age, diet, nutritional status, and environmental exposure, thus being able to characterize a given phenotype (Bouatra et al., 2013; Slupsky et al., 2007; Wahl et al., 2012). Urine production is a constant process and its collection is easy and non-invasive (Slupsky et al., 2007). Urine metabolome has been well explored in studies with adults (Slupsky et al., 2007; Yu et al., 2012; Holmes et al., 2008), but only a few studies have been conducted with children (Zhang et al., 2013; Chiu et al., 2016; McCormack et al., 2013). Considering this, analyzing urine from prepubertal children is very attractive for metabolomic studies, as it targets the molecular mechanisms behind childhood obesity and cardiometabolic alterations.

This study aimed to investigate if there is an amino acid fingerprint in the urine of obese prepubertal children and explore possible mechanisms through which the altered metabolites can contribute to obesity and cardiometabolic diseases.

## Materials and methods

### Participants and ethical aspects

This is a retrospective cross-sectional study conducted with a sample of 110 pre-pubescent children, both sexes, aged 9–10 years who participated in previous studies to evaluate cardiovascular health and nutritional status in children from public and private elementary schools in Vitória–ES (Brazil) (Batista et al., 2015). All procedures were according to ethical standards and approved by the Ethics and Research Committee on Human Experimentation from the Universidade Federal de São Paulo (register number: 16613619.1.0000.5505). Informed consent was obtained from parents or legal guardians before enrollment.

### Anthropometric measurements

Anthropometric parameters (weight, height, waist, and hip circumferences) were assessed following WHO recommendations described by Batista et al. (Batista et al., 2015). Briefly, body weight was determined using an electronic scale (Toledo®, model 2096, Brazil) with the participant standing barefoot wearing underwear only. The height was measured with a stadiometer (Seca®, model 206) attached to a flat wall. Using an anthropometric tape, the waist circumference was measured in the horizontal plane at the midpoint between the lowest rib and iliac crest (Sanny®).

Subjects were divided as normal weight (NW), overweight (OW), and obese (OB) according to their Body Mass Index classification for age and sex (BMI-for-age) using free AnthroPlus® software based on WHO growth reference (de Onis et al., 2007). Waist-to-hip (WHiR) (Freedman et al., 1999) and waist-to-height (WHtR) (Vieira et al., 2017) ratios were calculated using primary anthropometric data.

### Assessment of cardiovascular and biochemical parameters

Trained researchers conducted examinations in a controlled environment. Blood samples were collected under fasting conditions to measure urea, creatinine, uric acid, glucose, total cholesterol, LDL-cholesterol, HDL-cholesterol, VLDL-cholesterol, and triglycerides. All analyses were performed using commercial kits in a single laboratory (Central Laboratory of the Social Service of Industry, SESI-ES, Brazil). Blood pressure (BP) was measured in the left arm with an oscillometric device (OMROM® model HEM-705CP) using cuff size according to the manufacturer’s recommendations. Children were comfortably seated with their feet flat on the floor. Three BP readings were taken with 2-min intervals between each, and the average of the last two measurements was calculated for systolic BP (SBP), diastolic BP (DBP), and heart rate (HR) (Batista et al., 2015).

### Evaluation of urinary amino acids profile

A 12-h urine sample was collected from each participant and used for amino acid qualification and quantification. The concentration of thirty-nine amino acids was determined in urine samples by using the *Amino Acids Analysis Kit* (Zivak®, Turkey) by high-performance liquid chromatography (HPLC) coupled to an electrospray ionization (ESI) mass spectrometry (MS/MS) system. Regarding the chromatographic and mass spectrometry conditions, the analyses were strictly performed following the manufacturer’s protocol for the Amino Acid Biological Fluids LC-MS/MS Analysis Kit (Plasma and Serum and Urine and Cerebrospinal Fluid) (Ref: ZV-3002–0200–10), produced by ZIVAK. A Thermo Scientific (TSQ Quantum Access Max) was employed with a quaternary pump (Accela 600 pump model) with an automatic sampler and a triple quadrupole mass spectrometer analyzer. The concentration of all amino acids was corrected by the urinary creatinine concentration. The amino acids measured were: 3-methyl-histidine (3-MeHIS); 5-Hydroxy-L-tryptophan (5-HTRP); Alanine (ALA); Alpha-aminoadipic acid (AAA); Alpha-aminobutyric acid (ABA); Alpha-aminopimelic acid (APA); Anserine (ANS); Arginine (ARG); Asparagine (ASN); Aspartic Acid (ASP); Beta alanine (BALA); Beta-aminoisobutyric acid (BAIB); Carnosine (CAR); Citrulline (CIT); Cystathionine (CTH); Cysteine (CYS); Cystine (C-C); Gamma-aminobutyric acid (GABA); Glutamic Acid (GLU); Glutamine (GLN); Glycine (GLY); Histidine (HIS); Homocystine (HC-HC); Hydroxylysine (HYL); Hydroxyproline (HYP); Isoleucine (ILEU); Leucine (LEU); Lysine (LYS); 1-methyl-histidine (1-MeHIS); Ornithine (ORN); Phenylalanine (PHE); Proline (PRO); Sarcosine (SAR); Serine (SER); Thiaproline (THPR); Threonine (THR); Tryptophan (TRP); Tyrosine (TYR) and Valine (VAL). The handling of calibrants, controls, and samples, as well as the settings of the analytical methods, were carried out following the manufacturer’s instructions, and data were analyzed using Xcalibur version 2.0 software (Thermo Fisher Scientific). Amino acids whose concentrations were below the detection capability of equipment (such as Homocysteine, Taurine, N-acetyl-L-tyrosine, O-Phospho-L-serine, Histamine, Methionine, and Serotonin) were not included in the analysis. The CAS numbers for each amino acid are listed in Supplementary Table S1.

### Statistical analyses

All data are expressed as medians ± SEM (standard error of the median). Differences between groups were assessed using ANOVA followed by Tukey’s multiple comparison *post hoc* test, Kruskal-Wallis and Dunn’s Test or Mood’s median test, and Pairwise median test. The tests were chosen considering the variable’s distribution and variability according to indications in the table legends. Statistical analyses were performed with R, version 3.6.2 (R Core Team, 2019). A value of *p* ≤ 0.05 was considered to be statistically significant.

Raw metabolomic data were imported into the R software environment for preprocessing. Data normalization was performed using quotient normalization, and resulting data were log-transformed to stabilize variance and improve normality. Missing values were imputed using the k-nearest neighbor (k-NN) algorithm. Processed data were then analyzed using MetaboAnalystR (version 4.0) for comprehensive metabolomic data analysis. A volcano plot was generated to identify significantly different metabolites between the normal and excess weight (OW and OB) groups. Metabolites with a fold change (FC) greater than 1.25 and a p-value less than 0.05 were considered significant.

## Results

### Population characteristics

In this cohort, 54.5% of the participants were male and 45.5% were female. Regarding the nutritional status of the prepubescents, 40.9% showed normal weight, 19.1% were overweight and 40.0% were obese. All anthropometric data such as BMI-for-age (percentile and z-score), waist circumference, hip circumference, WHiR, and WHtR showed significant differences between the groups. As expected, there was a progressive increase in these parameters in the OW and OB groups compared to the NW group. Also, the OB group had higher height and height for age z-scores and percentiles than the NW group. These results are described in Table 1.

**TABLE 1:** Anthropometric parameters and growth indicators according to the nutritional status of prepubescent children. Numbers within parentheses indicate the sample size. kg = kilograms, m = meters, cm = centimeters, BMI = Body Mass Index; WHiR = waist-to-hip ratio; WHtR = waist-to-height ratio; NS = non-significant. ^#^Mood’s median test and Pairwise median test. *Kruskal-Wallis and Dunn’s Test. ¨ANOVA and Tukey HSD.^B^ Normal Weight ≠ Obesity;^C^ Overweight ≠ Obesity;^D^ All groups differ from each other.

Parameters/Indicators	Normal Weight (NW)	Overweight (OW)	Obesity (OB)	p-value
Sex				
Male	20.9%	6.4%	27.3%	*0,0282^C^
Female	20.0%	12.7%	12.7%	*NS
Age (years)	10.00 ± 0.09	9.00 ± 0.14	9.5 ± 0.10	*NS
Body Mass (kg)	31.14 ± 0.85	40.40 ± 1.44	50.13 ± 2.04	^#^< 0.0001^D^
Height (m)	1.41 ± 0.01	1.44 ± 0.02	1.43 ± 0.01	¨0.0116^B^
Percentile Height/a	64.70 ± 5.29	70.60 ± 7.96	84.85 ± 3.93	*0.0002^B^
*Z-score* Height/a	0.38 ± 0.18	0.54 ± 0.28	1.03 ± 0.16	¨0.0002^B^
BMI (kg/m^2^)	16.27 ± 0.25	19.91 ± 0.23	23.64 ± 0.61	^#^< 0.0001^D^
Percentile BMI-for-age	45.40 ± 4.63	92.40 ± 1.13	99.40 ± 020	^#^< 0.0001^D^
*Z-score* BMI-for-age	−0.06 ± 0.14	1.44 ± 0.07	2.51 ± 0.10	^#^< 0.0001^D^
Waist circumference (cm)	58.00 ± 0.85	69.50 ± 1.41	78.03 ± 1.57	^#^< 0.0001^D^
Hip circumference (cm)	71.50 ± 0.90	81.00 ± 1.38	88.05 ± 1.51	^#^< 0.0001^D^
WHiR	0.80 ± 0.01	0.84 ± 0.01	0.89 ± 0.01	*< 0.0001^D^
WHtR	0.41 ± 0.01	0.48 ± 0.01	0.55 ± 0.01	# < 0.0001^D^

Biochemical test values are described in Table 2, and only the uric acid levels significantly increased in the OB group compared to the NW group (4.10 vs. 3.30, p = 0.0013). As expected, the OW and OB groups have shown higher systolic (109.00 and 110.00 vs. 102.00, p = 0.0001) and diastolic (67.00 and 66.75 vs. 60.00, p < 0.0001) blood pressures; however, no differences were observed in heart rate.

**TABLE 2:** Distribution of cardiovascular parameters, and biochemical tests according to nutritional status. “u” = urine sample measurement SBP = systolic blood pressure; DBP = diastolic blood pressure; HR = heart rate; NS = non-significant. ^#^Mood’s median test and Pairwise median test. *Kruskal-Wallis and Dunn’s Test. ANOVA and Tukey HSD. (A) Normal Weight ≠ Overweight; (B) Normal Weight ≠ Obesity; (D) All groups differ from each other.

Parameter	Normal Weight (NW)	Overweight (OW)	Obesity (OB)	p-value
Blood tests				
Total cholesterol (mg/dL)	156.00 ± 6.90	173.00 ± 9.14	170.00 ± 6.52	¨NS
HDL cholesterol (mg/dL)	49.00 ± 2.31	50.00 ± 1.89	43.00 ± 2.05	#NS
LDL cholesterol (mg/dL)	94.00 ± 5.64	102.00 ± 7.09	99.40 ± 4.77	¨NS
VLDL cholesterol (mg/dL)	12.40 ± 1.32	15.60 ± 3.20	15.90 ± 2.21	#NS
Triglycerides (mg/dL)	59.00 ± 6.24	78.00 ± 16.00	80.00 ± 11.30	#NS
Uric acid (mg/dL)	3.30 ± 0.17	3.60 ± 0.16	4.10 ± 0.20	*0.0013^B^
Urea (mg/dL)	24.00 ± 1.41	24.00 ± 1.50	23.00 ± 1.37	¨NS
Creatinine (mg/dL)	0.70 ± 0.01	0.70 ± 0.02	0.70 ± 0.02	*NS
Glucose (mg/dL)	88.00 ± 1.58	90.00 ± 2.42	90.00 ± 1.79	*NS
Urine tests				
Sodium u (mEq/L)	110.50 ± 12.84	132.50 ± 22.78	159.00 ± 15.31	*NS
Potassium u (mEq/L)	20.15 ± 2.82	21.10 ± 5.10	26.40 ± 4.18	#NS
Creatinine u (mg/dL)	28.55 ± 3.05	20.61 ± 3.70	33.24 ± 3.83	*NS
Cardiovascular parameters				
SBP (mmHg)	102.00 ± 1.67	109.00 ± 2.52	110.00 ± 1.73	¨0.0001^A,B^
Percentile SBP	46.00 ± 4.97	65.00 ± 6.50	73.00 ± 4.56	*0.0005^A,B^
DBP (mmHg)	60.00 ± 1.60	67.00 ± 2.24	66.75 ± 1.40	*< 0.0001^A,B^
Percentile DBP	49.00 ± 3.74	69.00 ± 4.87	66.50 ± 3.82	*< 0.0001^A,B^
HR (bpm)	81.50 ± 2.20	80.50 ± 3.11	79.25 ± 1.91	*NS

### Urinary amino acids profile

In the amino acid analysis method, the limit of quantification (LOQ) is defined as the signal-to-noise ratio of 10. The results obtained for the LOQs showed a linear range of 0.01–0.32 ng mL−1 for AA with correlation coefficients (r) greater than 0.994 which can be seen in Supplementary Table S2.

**FIGURE 1:**
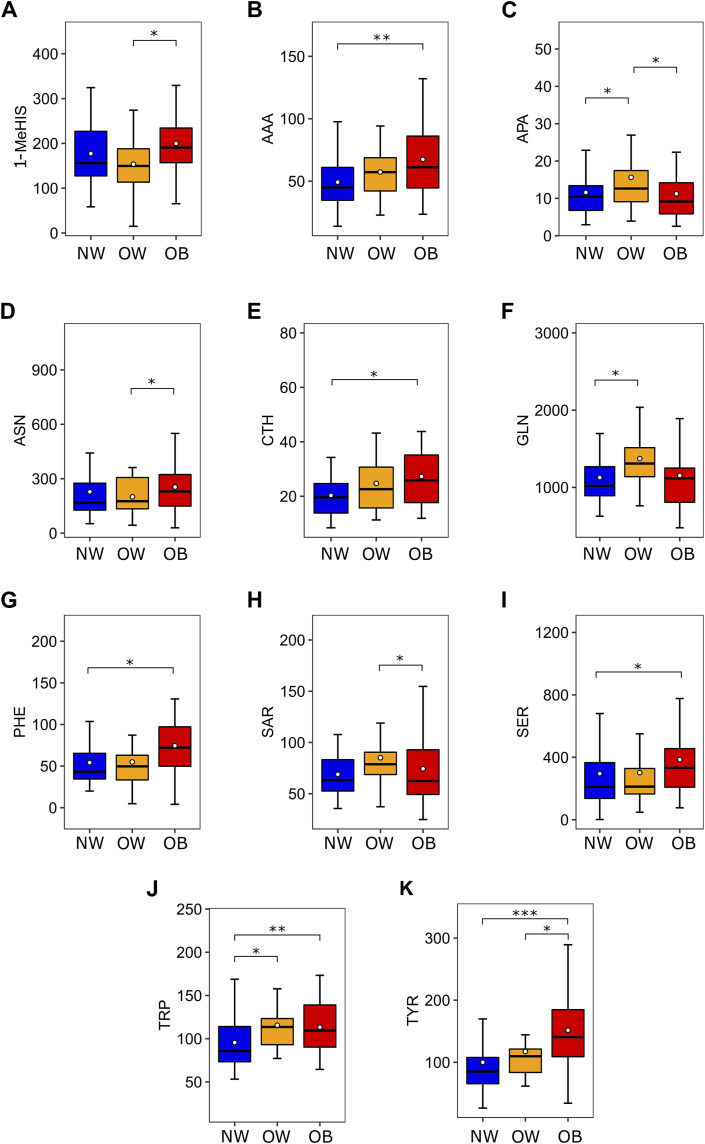
Amino acids which are differentially expressed according to nutritional status. **(A)** 1-MeHIS concentration in urine (nmol/mg of urinary creatinine) according to nutritional status. ANOVA (p = 0.047) followed by Tukey HSD (OW ≠ OB). **(B)** AAA concentration in urine (nmol/mg of urinary creatinine) according to nutritional status. Kruskal-Wallis (p < 0.01) followed by Benjamini-Hochberg (NW ≠ OB). **(C)** APA concentration in urine (nmol/mg of urinary creatinine) according to nutritional status. Mood’s Median Test (p = 0.046) followed by Pairwise median test (NW ≠ OW and OW ≠ OB). **(D)** ASN concentration in urine (nmol/mg of urinary creatinine) according to nutritional status. Mood’s Median Test (p = 0.049) followed by Pairwise median test (OW ≠ OB). **(E)** CTH concentration in urine (nmol/mg of urinary creatinine) according to nutritional status. Kruskal-Wallis (p = 0.015) followed by Benjamini-Hochberg (NW ≠ OB). **(F)** GLN concentration in urine (nmol/mg of urinary creatinine) according to nutritional status. Mood’s Median Test (p = 0.029) followed by Pairwise median test (NW ≠ OW). **(G)** PHE concentration in urine (nmol/mg of urinary creatinine) according to nutritional status. Kruskal-Wallis (p < 0.01) followed by Benjamini-Hochberg (NW ≠ OB). **(H)** SAR concentration in urine (nmol/mg of urinary creatinine) according to nutritional status. Mood’s Median Test (p = 0.024) followed by Pairwise median test (OW ≠ OB). **(I)** SER concentration in urine (nmol/mg of urinary creatinine) according to nutritional status. Kruskal-Wallis (p = 0.049) followed by Benjamini-Hochberg (NW ≠ OB). **(J)** TRP concentration in urine (nmol/mg of urinary creatinine) according to nutritional status. Kruskal-Wallis (p < 0.01) followed by Benjamini-Hochberg (NW ≠ OW and NW ≠ OB). **(K)** TYR concentration in urine (nmol/mg of urinary creatinine) according to nutritional status. Kruskal-Wallis (p < 0.001) followed by Benjamini-Hochberg (NW ≠ OW and NW ≠ OB). Post-hoc tests: *p < 0.05, **p < 0.01, and ***p < 0.001. Abbreviations: 1-MeHIS, 1-methyl-histidine; AAA, alpha-aminoadipic acid; APA, alpha-aminopimelic acid; ASN, asparagine, CTH, cystathionine; GLN, glutathione; PHE, phenylalanine; SAR, sarcosine; SER, serine; TRP, tryptophan; TYR, tyrosine; NW, normal weight; OW, overweight; OB, obesity.

Several amino acids showed relevant statistical differences according to nutritional status, as shown in Figure 1. Briefly, the concentration of the aromatic amino acids PHE, TRP, and TYR was significantly increased in the urine of OB group compared to the NW group (72.08 vs. 43.04, p < 0.05; 109.36 vs. 85.65, p < 0.01 and 140.54 vs. 85.07, p < 0.001, respectively), TYR levels were also higher in the OW group than NW group (109.74 vs. 85.07, p < 0.05) and TRP concentration was higher in OW group compared to NW group (113.55 vs. 85.65, p < 0.05). The OB group also presented augmented levels of AAA (61.18 vs. 44.89, p < 0.01), CTH (25.86 vs. 19.55, p < 0.05) and SER (332.40 vs. 210.97, p < 0.05) compared to NW group and increased concentration of urinary 1-MeHIS compared to OW group (190.62 vs. 149.59, p < 0.05). The most expressive concentrations of the amino acids APA, GLN, and SAR were found in the OW group. APA levels were higher in OW compared to NW and OB groups (12.64 vs. 10.45, p < 0.05 and 12.64 vs. 9.17, p < 0.05). Urinary concentration of GLN was higher in OW than OB group (1309.16 vs. 1024.30, p < 0.05) while SAR levels were increased in OW compared to OB group (78.75 vs. 62.36, p < 0.05). A table evaluating all 39 amino acids according to nutritional status is provided as supplementary material (Supplementary Table S3).

**FIGURE 2:**
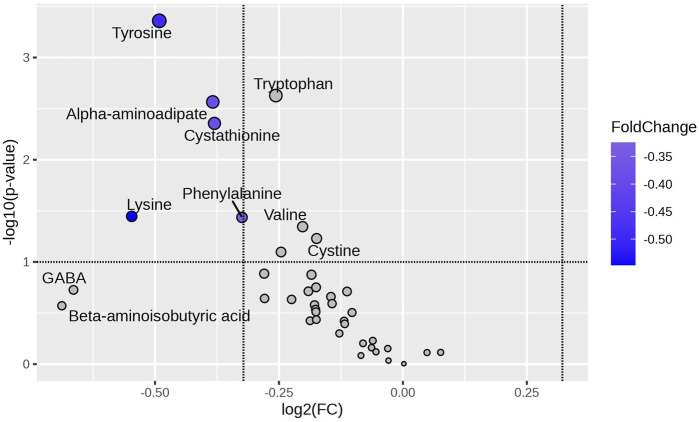
Amino Acid Variability Between NW and OW + OB Groups. The Volcano plot highlights amino acids differentially abundant between Normal Weight (NW) and Excess Weight (Overweight + Obesity; OW + OB) groups, using the NW group as the reference. Amino acids with a log2 fold change (FC) of less than −2 and a -log10(p) value greater than 1.3 (p-value <0.05) were considered significantly downregulated in the NW group compared to the OW + OB group. The amino acids phenylalanine, tyrosine, α-aminoadipic acid, cystathionine, and lysine fall within this category, indicating higher abundance in the OW + OB group. Blue highlights indicate amino acids with a p-value <0.05. FC: Fold Change.

Figure 2 presents a metabolomic approach to demonstrate the relationship between the statistical significance of an amino acid expression and the magnitude of the change in its expression (considering the mean). For this analysis, we considered two groups: excess weight (OW and OB) and normal weight (NW). Five metabolites (PHE, CTH, AAA, TYR, and LYS) presented significant fold change between the groups, being more expressed in the excess of weight group. The most expressive magnitude of fold change was observed in the following order: LYS, TYR, AAA, CTH, and PHE. TYR exhibited the most significant statistical difference between groups. Supplementary Table S4 shows the fold change of the mean for the normal weight group compared to the excess weight group for all amino acids evaluated in the study (Supplementary Table S4).

Regarding Principal Component Analysis (PCA), PC1 accounts for 78.3% of the variance, while PC2 explains 15.7%. Despite PC1’s high variance, its loadings cluster near the origin, indicating no strong driver of separation. Moreover, metabolites furthest from the origin in PC1 do not align with those statistically significant in the Volcano Plot, suggesting that separation arises from minor cumulative changes rather than distinct metabolic shifts. PC2 captures additional variation but remains limited. Thus, while statistical differences exist, PCA alone does not fully capture metabolic shifts, highlighting the need for complementary analyses (Supplementary Figure S1).

## Discussion

Research groups and health organizations worldwide are unanimous in warning about the rapid increase in overweight and obesity prevalence and its consequences for individuals’ and communities’ health (Vieira et al., 2017; GBD, 2015 Obesity Collaborators et al., 2017; NCD Risk Factor Collaboration NCD-RisC, 2017). These consequences are directly related to higher health service costs, a high risk of disease development, and related mortality (Di Angelantonio et al., 2016; GBD, 2015 Obesity Collaborators et al., 2017; NCD Risk Factor Collaboration NCD-RisC, 2017).

Weight and BMI are anthropometric measures that reflect the quality of nutrition and healthiness of the living environment during childhood and adolescence. These parameters are good predictors of health and developmental outcomes throughout life (Park et al., 2012; Tanner, 1987). As expected, all anthropometric measurements were progressively increased in the groups OW and OB, as shown in Table 1.

More important than total body fat, WHtR and WHiR predict fat distribution in the upper part of the body around the abdomen, which is associated with metabolic changes. WHiR and WHtR values of OW (0.84 ± 0.01; 0.48 ± 0.01, p < 0.0001) and OB (0.89 ± 0.01; 0.55 ± 0.01, p < 0.0001) groups, respectively, compared to NW (0.80 ± 0.01; 0.41 ± 0.01) suggest a progressive increase in abdominal fat in greater degrees of excess weight. Zeng et al. (Zeng et al., 2010) looking for biomarkers suggested that the waist-hip ratio, together with total triglycerides, total cholesterol, high-density lipoprotein, and low-density lipoprotein are the most critical parameters that correlate with the metabolic disturbances of childhood obesity (Lo et al., 2016). In a systematic review of thirty-nine studies, Park et al. (Park et al., 2012) observed evidence for associations between childhood BMI and type 2 diabetes, hypertension, and coronary heart disease, as in other studies (Lo et al., 2016; Libert et al., 2018; Juonala et al., 2011; Liu et al., 2021). Moreover, uric acid levels are substantially increased in obese subjects and proportionally associated with BMI (Weihrauch-Blüher et al., 2023), as observed in our results.

Metabolomics has been used to study the metabolic signature of obesity. It describes differential responses to dietary interventions, predicts health outcomes, and allows the study of the effects of specific nutritional patterns on obesity-related metabolites (Zhang et al., 2013; Rangel-Huerta et al., 2019; Szczerbinski et al., 2022). These biomarkers can represent disease diagnostic tools for developing new therapeutic protocols (Hameed et al., 2020; Zhang et al., 2013).

Investigation of serum metabolite concentrations in obese children may lead to new insights into biological mechanisms associated with childhood obesity, for example, branch-chained amino acids and various lipid metabolites, including phosphatidylcholines, cholesteryl esters, triglycerides (Zhang et al., 2013; Szczerbinski et al., 2022). Oberbach et al. (Butte et al., 2015) identified 163 serum metabolites, 12 of which were significantly related to obesity. Among those, GLY, GLN, and glycerophosphatidylcholine 42:0 (PCaa 42:0) serum concentrations were higher, whereas PCaa 32:0, PCaa 32:1, and PCaa 40:5 were decreased in the obesity group compared to the normal weight group. Wahl et al. (Wahl et al., 2012) analyzed serum samples from obese and normal-weight children aged six to 15. Fourteen metabolites and 69 metabolite ratios were significantly different in obese children compared to normal-weight children. Butte et al. (Butte et al., 2015) observed that obese Hispanic children had increased plasma concentrations of LEU, ILEU, and VAL but lower ASN, ASP, GLY, and SER concentrations. Plasma amino acid profile has shown a strong correlation with nutritional status (McCormack et al., 2013; Butte et al., 2015; Wu et al., 2024; Morris et al., 2012).

Despite urine being a promising biological fluid for metabolomics research, few studies have been conducted with this fluid compared to the numerous studies conducted with blood samples. However, urinary metabolomics research focuses on characterizing the metabolic profile present in urine, providing invaluable insights into both physiological and pathological processes. This comprehensive analysis facilitates the discovery of biomarkers for disease diagnosis, treatment monitoring, and elucidation of metabolic pathways (Scalabre et al., 2017; Szczerbinski et al., 2022; Bouatra et al., 2013; Cho et al., 2017; Chavira-Suárez et al., 2020). Detecting metabolites in prepubertal urine represents a gap in the existing literature, and your investigation provides additional insights.

An amino acid signature characteristic of OB group was found in our study, with elevated levels of AAA, ASN, CTH, 1-MeHIS, SER, and aromatic amino acids, and reduced concentrations of APA, and SAR. The OW group presented most expressive urinary levels of APA, GLN, and SAR.

Urinary excretion of 3-MeHIS indicates protein catabolism, as it comes from skeletal muscle actin and myosin, and is also directly related to meat consumption (Cross et al., 2011). The higher levels of 1-MeHIS observed in the OB group may reflect dietary differences in meat intake. A controlled feeding study investigated 1-MeHIS and 3-MeHIS as potential biomarkers of meat intake and found a dose-dependent association between meat intake and urinary excretion of 1- and 3-MeHIS (Cross et al., 2011; Cross et al., 2014).

Our results show increased values of CTH and SER for the OB group. One-carbon metabolism is a metabolic network driven by three interrelated metabolic pathways: the folate cycle, the homocysteine-methionine cycle, and the transsulfuration pathway. If there is abundant methionine, the transsulfuration pathway will become active, by which homocysteine reacts with serine to form cystathionine by cystathionine β-synthase (Zhu et al., 2024; Dwight, 2020). Due to the inability to quantify homocysteine and methionine, their contribution to the increased levels of these other amino acids cannot be directly determined. Nonetheless, elevated levels of CTH and SER may indicate enhanced methionine metabolism and, consequently, increased homocysteine production through cystathionine β-synthase action (Dwight, 2020; Zhang et al., 2020). On the other hand, Butte et al. (Wu et al., 2024) found reduced serum levels of SER, which were associated with risk factors for insulin resistance, hypertriglyceridemia, and hyperuricemia.

Increased levels of AAA were observed in the OB group, as reported by Libert, Nowacki, and Natowicz (Libert et al., 2018), who also found elevated levels in adult subjects with obesity and diabetes. AAA can be generated by LYS metabolism. L-lysine is first converted to saccharopine by condensation with α-ketoglutarate, which is then reduced to 2-aminoadipic semialdehyde, releasing GLU. Subsequently, 2-aminoadipic semialdehyde is interconverted to AAA (Dwight, 2020). Catabolism of AAA forms 2-ketoadipic acid, and in TRP catabolism, it also occurs through L-kynurenine degradation (Dwight, 2020). High values of AAA and TRP, but not LYS, may suggest changes in these metabolic pathways in the OB group. Wang et al. (Wang et al., 2013) observed that AAA predicted the development of diabetes in normoglycemic individuals and hypothesized that AAA levels were increased in response to hyperglycemia, increasing insulin secretion and contributing to a compensatory mechanism to maintain glucose homeostasis in early insulin resistance. It also suggests that AAA is a marker of diabetes risk and a potential modulator of glucose homeostasis in humans. Additional investigations should be done to link AAA to weight gain, insulin resistance, and T2DM.

In addition to AAA, GLN has also been linked to insulin resistance (Cheng et al., 2012; Newgard et al., 2009). Hanzu et al. (Hanzu et al., 2014) found high levels of GLN and ALA in the visceral adipose tissue of individuals with obesity. Due to the high gluconeogenic effect, the increased amount of these amino acids released by visceral adipose tissue contributes to hyperinsulinemia and the development of insulin resistance (Hanzu et al., 2014). Higher GLN levels in the OW group suggest that being overweight may modify glucose metabolism due to increased body fat, especially visceral fat (Payab et al., 2021; Yan et al., 2023), evidenced by the augmented waist and hips circumferences and relative ratios. Despite the evidence, other groups have observed reduced levels of GLN in serum samples from children and urine samples from obese adolescents (Wahl et al., 2012; Cho et al., 2017).

Furthermore, it is well established that branched-chain and aromatic amino acids are indicators of the development of insulin resistance in normoglycemic young adults. This fact indicates a strong association between amino acids (Singh et al., 2023), particularly aromatics (TRP, PHE, and TYR), and the risk of future development of diabetes mediated partially by insulin resistance (Rangel-Huerta et al., 2019; Newgard et al., 2009; Martos-Moreno et al., 2005).

Under conditions of obesity, most studies demonstrate significant changes in blood and urinary values of aromatic (Butte et al., 2015; Cho et al., 2017; Haufe et al., 2016) and branched-chain amino acids (Szczerbinski et al., 2022; Butte et al., 2015; Bagheri et al., 2018). TRP, PHE, and TYR levels were elevated in the OB group, as other studies have also shown an association between increased concentrations of aromatic amino acids and obesity in both younger and older children, with different metabolic disturbances involved in the progression from overweight to obesity between the two age groups (Butte et al., 2015; Wu et al., 2024; Newgard et al., 2009; Payab et al., 2021; Bagheri et al., 2018; Kim et al., 2010; Yu et al., 2018).

Exploring amino acids through Volcano Plot analysis comparing children with normal weight (NW) and those with excess weight (OW + OB), using the NW group as a reference, the amino acids that exhibited a log2 fold change of less than −2 and -log10(p) of less than 1.3 — phenylalanine, tyrosine, α-aminoadipic acid, cystathionine, and lysine — showed greater significance and were negatively regulated in the NW group. Notably, tyrosine emerged as the amino acid with the most significant changes and the most considerable magnitude of change.

Tyrosine levels were related to increased hepatic fat content, suggesting hepatic dysfunction associated with a metabolic disorder (Libert et al., 2018; Haufe et al., 2016). In addition, tyrosine contributes to the profile described in obese children (Payab et al., 2021). It may be a possible predictor of insulin resistance in these children and the most sensitive metabolite for the classification of obesity (Handakas et al., 2022; Butte et al., 2015; Martos-Moreno et al., 2005). Increased plasma levels of phenylalanine and tyrosine have been observed in most analyses of amino acid biomarkers in obesity and T2DM (Payab et al., 2021; Haufe et al., 2016; Zhao et al., 2016; Park et al., 2015). In this regard, elevated levels of PHE and its hydroxylation product, TYR, provided strong relevance as biomarker metabolites predictive of the development of cardiovascular disease and diabetes type 2. Suzuki et al. (2019) described that a state of unbalanced or increased amino acids associated with obesity may exacerbate obesity and insulin sensitivity (Suzuki et al., 2019).

Several limitations of the present study need consideration. The relatively small size of groups may have interfered with statistical significance. Moreover, our study used BMI-for-age to classify obesity, rather than body fat content and distribution, which would be a more accurate parameter of adiposity and metabolic changes. However, determining criteria for cutting off body fat in children remains under discussion in the pediatric area. Additionally, other children’s data should be compared with these results to trace the relationship between nutritional status and the metabolic profile of amino acids. Our working group is already outlining new data crossings.

## Conclusion

The search for a biomarker that indicates the development of obesity is essential in a population that has not yet reached the reproductive stage. Since it allows blocking the progression of obesity and related comorbidities from measures that encourage healthier habits, before the need for drug therapies. Analysis of amino acids in urine through metabolomics showed a strong association between childhood obesity and increased levels of AAA, CTH, SER, and aromatic amino acids, particularly TYR, which appears to be a good candidate for obesity biomarkers. Potential mechanisms for increased levels of these amino acids include increasing protein degradation and impairment of oxidative metabolism in some tissues. Additional investigations must be done to determine whether the metabolism of TYR and other aromatic amino acids could identify the metabolic profile of children with obesity and other disorders.

## Data Availability

The original contributions presented in the study are included in the article/Supplementary Material, further inquiries can be directed to the corresponding author.
