# Correction: Urinary amino acid metabolomic profiling and its association with childhood obesity in prepubescent individuals

**DOI:** 10.3389/fphys.2025.1703729

**Published:** 2025-10-07

**Authors:** Mariana Doce Passadore, Nayara Azinheira Nobrega Cruz, Mariana Zuccherato Bocato, Leonardo de Abreu Ferreira, Marcelo Yudi Icimoto, Maria del Carmen Bisi Molina, José Geraldo Mill, Fernando Barbosa Junior, Dulce Elena Casarini, Lilian Caroline Gonçalves de Oliveira

**Affiliations:** ^1^ Postgraduation Program of Nephrology, Nephrology Division, Department of Medicine, Universidade Federal de São Paulo (UNIFESP/EPM), Sao Paulo, Brazil; ^2^ Postgraduation Program of Translational Medicine, Nephrology Division, Department of Medicine, Universidade Federal de São Paulo (UNIFESP/EPM), Sao Paulo, Brazil; ^3^ Analytical and System Toxicology Laboratory, Department of Clinical, Toxicological and Bromatological Analysis, Faculty of Pharmaceutical Sciences of Ribeirao Preto, Universidade de São Paulo, Sao Paulo, Brazil; ^4^ Universidade Presbiteriana Mackenzie, Sao Paulo, Brazil; ^5^ Department of Biophysics, Universidade Federal de São Paulo (UNIFESP/EPM), Sao Paulo, Brazil; ^6^ Department of Integrated Health Education, Center for Health Sciences, Universidade Federal do Espírito Santo, Vitória, Brazil; ^7^ Department of Physiological Sciences, Universidade Federal do Espírito Santo, Vitória, Brazil; ^8^ Nephrology Division, Department of Medicine, Universidade Federal de São Paulo (UNIFESP/EPM), Sao Paulo, Brazil

**Keywords:** childhood obesity, prepuberty, urinary amino acids, metabolomics, biomarkers

Incorrect funding

In the published article, the funder FAPESP (grant number 2025/06905-2, to Lilian Caroline Gonçalves de Oliveira) was erroneously omitted.

The correct **Funding** statement is below.

The author(s) declare that financial support was received for the research and/or publication of this article. This study was financially supported by Fundação de Amparo à Pesquisa do Estado de São Paulo (FAPESP) (process number 2018/23953-7), which was the primary funding source, and Espírito Santo Research Foundation (FAPES) (process number 65854420/2014). Additional support was provided by FAPESP (process numbers 2025/06905-2, 2024/07709-0, 2019/16955-6, 2018/24069-3) and CNPq (process number 406442/2022-3).

**Author name**

In the published article, author “Lilian Caroline Gonçalves de Oliveir” name was spelt incorrectly. The correct name is Lilian Caroline Gonçalves de Oliveira.

The original article has been updated.

