# Supplementary material for: Urinary amino acid metabolomic profiling and its association with childhood obesity in prepubescent individuals
Source: Front Physiol. 2025 Apr 29;16:1524939. doi: 10.3389/fphys.2025.1524939 (PMC12069889; doi:10.3389/fphys.2025.1524939)

**Supplementary material**

**Table S1.** Abbreviations and CAS Numbers of the analyzed amino acids

| Amino Acid | CAS Number |
| --- | --- |
| 3-Methyl-histidine (3-MeHIS) | 368-16-1 |
| 5-Hydroxy-L-tryptophan (5-HTRP) | 4350-09-8 |
| Alanine (ALA) | 56-41-7 |
| Alpha-aminoadipic acid (AAA) | 542-32-5 |
| Alpha-aminobutyric acid (ABA) | 1492-24-6 |
| Alpha-aminopimelic acid (APA) | 627-76-9 |
| Anserine (ANS) | 584-85-0 |
| Arginine (ARG) | 74-79-3 |
| Asparagine (ASN) | 70-47-3 |
| Aspartic acid (ASP) | 56-84-8 |
| Beta-alanine (BALA) | 107-95-9 |
| Beta-aminoisobutyric acid (BAIB) | 144-90-1 |
| Carnosine (CAR) | 305-84-0 |
| Citrulline (CIT) | 372-75-8 |
| Cystathionine (CTH) | 56-88-2 |
| Cysteine (CYS) | 52-90-4 |
| Cystine (C-C) | 56-89-3 |
| Gamma-aminobutyric acid (GABA) | 56-12-2 |
| Glutamic acid (GLU) | 56-86-0 |
| Glutamine (GLN) | 56-85-9 |
| Glycine (GLY) | 56-40-6 |
| Histidine (HIS) | 71-00-1 |
| Homocystine (HC-HC) | 626-72-2 |
| Hydroxylysine (HYL) | 1190-74-9 |
| Hydroxyproline (HYP) | 51-35-4 |
| Isoleucine (ILEU) | 73-32-5 |
| Leucine (LEU) | 61-90-5 |
| Lysine (LYS) | 56-87-1 |
| 1-Methyl-histidine (1-MeHIS) | 332-80-9 |
| Ornithine (ORN) | 70-26-8 |
| Phenylalanine (PHE) | 63-91-2 |
| Proline (PRO) | 147-85-3 |
| Sarcosine (SAR) | 107-97-1 |
| Serine (SER) | 56-45-1 |
| Thiaproline (THPR) | 45521-09-3 |
| Threonine (THR) | 72-19-5 |
| Tryptophan (TRP) | 73-22-3 |
| Tyrosine (TYR) | 60-18-4 |
| Valine (VAL) | 72-18-4 |

| **Table S2.** Linearity and limits of quantification for analysis of 47 amino acids. | | | | | |
| --- | --- | --- | --- | --- | --- |
| **Amino acids** | **Linear range**  **(ng mL**^–^**^1^)** | **Linear equation** | **r** | **LOQ**  **(ng mL**^–^**^1^)** | **CV**  **(%)** |
| 3–Methylhistidine | 43.2–282.0 | 0.7857+0.9987x | 0.999 | 2.10 | 3 |
| 5–Hydroxy-L-tryptophan | 4.0–11.8 | 1.6972+0.7745x | 0.998 | 0.08 | 4 |
| Alanine | 399.0–1111.0 | –1.1139+0.2509x | 0.998 | 13.4 | 7 |
| Alpha-Aminoadipic Acid | 13.5–47.0 | 0.6874+0.9974x | 0.997 | 0.10 | 5 |
| Alpha-Aminobutyric Acid | 14.3–50.4 | 0.8875+1.0842x | 0.998 | 0.05 | 10 |
| Alpha-Aminopimelic Acid | 5.6–20.2 | –0.2691+0.6572x | 0.998 | 0.02 | 6 |
| Anserine | 2.4–57.0 | 0.9987+0.2654x | 0.995 | 0.04 | 9 |
| Asparagine | 61.3–207.0 | 0.8816+1.1327x | 0.996 | 1.02 | 3 |
| Arginine | 37.8–152.6 | 0.6971+0.0987x | 0.997 | 0.81 | 1 |
| Aspartic Acid | 11.4–63.3 | 0.1369+0.1547x | 0.997 | 0.02 | 6 |
| Beta-Alanine | 25.0–141.4 | –1.3369+0.1246x | 0.998 | 0.12 | 11 |
| Beta-Aminoisobutyric Acid | 11.68–54.11 | 0.6974+0.3366x | 0.999 | 0.47 | 4 |
| Carnosine | 0.264–2.242 | 0.8872+0.4515x | 0.995 | 0.06 | 8 |
| Citrulline | 60.3–193.0 | 1.1801+1.8744x | 0.999 | 8.11 | 5 |
| Creatinine | 76.3–210.0 | 0.1130+0.0987x | 0.999 | 4.09 | 8 |
| Cystathionine | 12.0–46.0 | 0.8714+0.2216x | 0.997 | 0.12 | 8 |
| Cystine | 71.62–904.7 | –0.7167+0.2479x | 0.998 | 7.07 | 10 |
| Gamma-Aminobutyric Acid | 12.25–42.22 | 0.3032+0.2179x | 0.996 | 0.08 | 6 |
| Glutamic Acid | 101.8–269.1 | 0.1475+0.7813x | 0.997 | 10.02 | 7 |
| Glutamine | 467.0–1153.0 | 0.2577+0.8434x | 0.998 | 20.33 | 5 |
| Glycine | 186.5–744.0 | 0.9332+1.1870x | 0.997 | 10.02 | 4 |
| Histamine | 5.63–25.2 | 0.5942+0.0987x | 0.998 | 0.08 | 8 |
| Histidine | 75.60–302.0 | –0.0947+0.2274x | 0.999 | 0.70 | 7 |
| Hydroxylysine | 8.40–44.3 | 0.2679+1.6130x | 0.997 | 0.05 | 9 |
| Homocystine | 2.46–31.7 | 0.1517+0.4847x | 0.996 | 0.04 | 5 |
| Hydroxyproline | 19.6–64.5 | 1.4798+0.1471x | 0.998 | 0.17 | 3 |
| Isoleucine | 89.5–252.0 | 0.6693+0.8874x | 0.994 | 5.05 | 4 |
| Leucine | 253.0–680.0 | 0.2174+2.1813x | 0.998 | 10.07 | 6 |
| Lysine | 158.0–452.0 | 2.0974+0.2214x | 0.998 | 0.79 | 10 |
| Methionine | 22.65–121.0 | –0.0974+0.3697x | 0.997 | 0.06 | 9 |
| N-Acetyl-L-Tyrosine | 367.0–710.0 | 0.4515+1.8722x | 0.999 | 0.32 | 5 |
| N-Methylhistidine | 18.1–82,1 | 0.2974+0.0974x | 0.998 | 0.04 | 2 |
| Ornithine | 111.1–324.0 | 0.8863+0.8410x | 0.997 | 6.10 | 3 |
| O-Phospho-L-Serine | 41.43–232.0 | 0.7460+0.0741x | 0.997 | 1.16 | 12 |
| Phenylalanine | 55.1–210.4 | 1.1911+0.4233x | 0.996 | 0.05 | 4 |
| Proline | 248.0–730.0 | 0.8870+0.2416x | 0.998 | 12.11 | 8 |
| Sarcosine | 33.34–109.4 | 0.3271+0.4790x | 0.997 | 0.03 | 5 |
| Serine | 103.2–408.9 | –1.5515+0.6301x | 0.999 | 5.15 | 9 |
| Serotonin | 41.30–158.9 | 0.0974+2.3251x | 0.996 | 0.94 | 6 |
| Thiaproline | 21.1–74.7 | 1.4974+0.2317x | 0.998 | 0.05 | 7 |
| Threonine | 108.4–433.0 | 0.3251+0.0496x | 0.997 | 2.08 | 6 |
| Tryptophan | 43.1–137.0 | 0.6657+0.9974x | 0.997 | 0.42 | 7 |
| Tyrosine | 61.3–225.0 | 0.3693+0.2040x | 0.994 | 0.95 | 8 |
| Valine | 220.0–737.0 | 0.09145+0.4744x | 0.995 | 18.07 | 4 |
| Taurine | 192.0̄–824.0 | 0.2119+0.9831x | 0.996 | 0.24 | 12 |
| Cysteine | 59.10–105.43 | 0.7962+0.2864x | 0.997 | 0.58 | 11 |
| Homocysteine | 48.38–231.25 | 0.2387+0.2217x | 0.996 | 0.21 | 9 |

*LOQ: Limit of Quantification; CV: Coefficient of Variation

**Table S3.** Urinary amino acid concentrations (nmol/mg creatinine) according to nutritional status. nmol/mg CR = nmol/mg of urinary creatinine, NS = non-significant. ^#^Mood’s median test and Pairwise median test. *Kruskal-Wallis e Dunn’s Test. ¨ANOVA e Tukey HSD. ^A^ Normal Weight ≠ Overweight; ^B^ Normal Weight ≠ Obesity; ^C^ Overweight ≠ Obesity.

| Amino acid  (nmol/mg CR) | Normal Weight  (n = 45) | Overweight  (n = 21) | Obesity  (n = 44) | p-value |
| --- | --- | --- | --- | --- |
| 3-MeHIS | 360.25 ± 142.92 | 349.25 ± 137.39 | 539.54 ± 149.49 | *NS |
| 5-HTRP | 15.30 ± 0.84 | 14.60 ± 1.19 | 16.68 ± 0.86 | **¨**NS |
| ALA | 306.83 ± 23.50 | 303.05 ± 25.26 | 343.31 ± 32.06 | #NS |
| AAA | 44.89 ± 3.95 | 57.22 ± 5.89 | 61.18 ± 5.62 | *0.0065^B^ |
| ABA | 11.79 ± 1.270 | 13.67 ± 2.17 | 15.06 ± 1.22 | *NS |
| APA | 10.45 ± 1.24 | 12.64 ± 3.01 | 9.17 ± 1.48 | #0.0462^A,C^ |
| ANS | 82.59 ± 8.18 | 104.35 ± 21.66 | 63.23 ± 13.80 | #NS |
| ARG | 212.51 ± 24.78 | 305.33 ± 63.49 | 158.62 ± 41.20 | *NS |
| ASN | 168.53 ± 31.26 | 176.14 ± 26.24 | 229.87 ± 26.43 | #0.0493^C^ |
| ASP | 17.69 ± 1.26 | 20.95 ± 1.49 | 18.72 ± 0.98 | *NS |
| BALA | 17.65 ± 5.03 | 9.49 ± 5.88 | 15.90 ± 8.76 | #NS |
| BAIB | 67.63 ± 92.49 | 84.72 ± 252.70 | 106.11 ± 43.04 | #NS |
| CAR | 187.58 ± 37.09 | 170.54 ± 53.21 | 165.67 ± 52.45 | #NS |
| CIT | 94.46 ± 8.98 | 131.20 ± 22.37 | 88.25 ± 14.44 | #NS |
| CTH | 19.55 ± 1.63 | 22.60 ± 3.11 | 25.86 ± 2.36 | *0.0148^B^ |
| CYS | 70.82 ± 4.73 | 84.05 ± 12.81 | 56.60 ± 7.77 | #NS |
| C-C | 182.10 ± 8.61 | 202.70 ± 20.83 | 177.99 ± 14.30 | #NS |
| GABA | 97.82 ± 64.37 | 142.40 ± 196.52 | 117.60 ± 34.79 | #NS |
| GLU | 90.94 ± 7.60 | 123.42 ± 15.40 | 81.31 ± 10.44 | *NS |
| GLN | 1024.30 ± 185.89 | 1309.16 ± 135.83 | 1117.35 ± 90.62 | #0.0293^A^ |
| GLY | 1690.42 ± 203.95 | 1764.66 ± 252.50 | 1723.68 ± 224.46 | *NS |
| HIS | 1136.97 ± 136.23 | 1171.70 ± 103.48 | 1459.04 ± 111.40 | #NS |
| HC-HC | 5.15 ± 0.46 | 7.21 ± 1.34 | 4.46 ± 0.82 | #NS |
| HYL | 10.65 ± 1.12 | 16.73 ± 1.99 | 10.54 ± 2.22 | #NS |
| HYP | 5.05 ± 0.42 | 6.57 ± 1.07 | 4.47 ± 0.47 | #NS |
| ILEU | 53.86 ± 3.59 | 73.23 ± 9.27 | 54.76 ± 5.87 | #NS |
| LEU | 307.62 ± 24.43 | 412.81 ± 62.90 | 298.05 ± 40.79 | #NS |
| LYS | 165.56 ± 28.59 | 143.62 ± 23.96 | 229.76 ± 56.54 | #NS |
| 1-MeHIS | 155.73 ± 12.46 | 149.59 ± 18.14 | 190.62 ± 14.60 | **¨**0.0468^C^ |
| ORN | 16.46 ± 1.17 | 19.32 ± 1.28 | 18.36 ± 1.52 | #NS |
| PHE | 43.04 ± 5.66 | 49.66 ± 8.54 | 72.08 ± 7.15 | *0.0076^B^ |
| PRO | 269.27 ± 25.72 | 369.76 ± 65.07 | 235.76 ± 41.90 | #NS |
| SAR | 63.06 ± 4.62 | 78.75 ± 10.28 | 62.36 ± 7.42 | #0.0241^C^ |
| SER | 210.97 ± 51.93 | 212.26 ± 73.58 | 332.40 ± 48.39 | *0.0491^B^ |
| THPR | 72.79 ± 6.32 | 98.12 ± 16.40 | 67.66 ± 10.48 | #NS |
| THR | 271.64 ± 46.03 | 294.28 ± 21.34 | 317.18 ± 34.30 | #NS |
| TRP | 85.65 ± 5.66 | 113.55 ± 8.08 | 109.36 ± 6.05 | *0.0018^A,B^ |
| TYR | 85.07 ± 10.21 | 109.74 ± 14.42 | 140.54 ± 11.37 | *<0.0001^B^ |
| VAL | 100.58 ± 5.11 | 117.43 ± 12.51 | 100.53 ± 8.27 | #NS |

**Table S4.** Amino Acids Fold change (FC) values

| Amino Acid | Log 2 of Fold Change |
| --- | --- |
| 3-Methyl-histidine (3-MeHIS) | 0,09 |
| 5-Hydroxy-L-tryptophan (5-HTRP) | -0,02 |
| Alanine (ALA) | -0,13 |
| Alpha-aminoadipic acid (AAA) | -0,34 |
| Alpha-aminobutyric acid (ABA) | -0,24 |
| Alpha-aminopimelic acid (APA) | -0,21 |
| Anserine (ANS) | -0,25 |
| Arginine (ARG) | -0,27 |
| Asparagine (ASN) | 0,01 |
| Aspartic acid (ASP) | -0,13 |
| Beta-alanine (BALA) | 0,00 |
| Beta-aminoisobutyric acid (BAIB) | -1,01 |
| Carnosine (CAR) | 0,07 |
| Citrulline (CIT) | -0,23 |
| Cystathionine (CTH) | -0,36 |
| Cysteine (CYS) | -0,18 |
| Cystine (C-C) | -0,18 |
| Gamma-aminobutyric acid (GABA) | -0,95 |
| Glutamic acid (GLU) | -0,17 |
| Glutamine (GLN) | 0,00 |
| Glycine (GLY) | -0,04 |
| Histidine (HIS) | -0,09 |
| Homocystine (HC-HC) | -0,31 |
| Hydroxylysine (HYL) | -0,24 |
| Hydroxyproline (HYP) | -0,13 |
| Isoleucine (ILEU) | -0,23 |
| Leucine (LEU) | 0,24 |
| Lysine (LYS) | -0,40 |
| 1-Methyl-histidine (1-MeHIS) | 0,00 |
| Ornithine (ORN) | -0,07 |
| Phenylalanine (PHE) | -0,26 |
| Proline (PRO) | -0,24 |
| Sarcosine (SAR) | -0,21 |
| Serine (SER) | -0,22 |
| Thiaproline (THPR) | -0,24 |
| Threonine (THR) | 0,04 |
| Tryptophan (TRP) | -0,26 |
| Tyrosine (TYR) | -0,43 |
| Valine (VAL) | -0,23 |

Figure S1. Principal Component Analysis (PCA) analysis. Group 1 represents the obese + overweight and Group 2 represents the eutrophic patients. Principal Component 1 (PC1) accounts for 78.3% of the total variance, while PC2 explains 15.7%. Further details are provided in the main text.


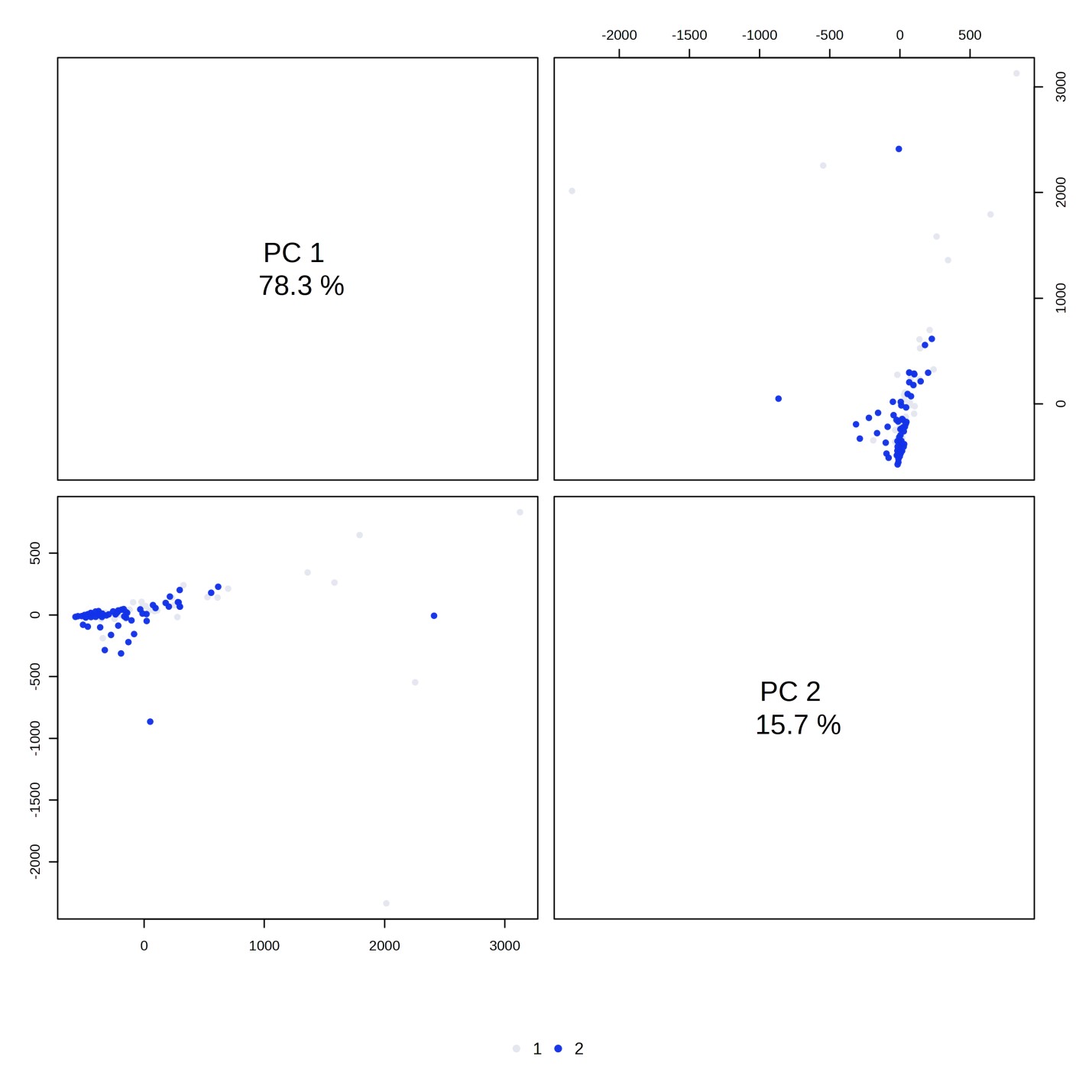

Supplement: Supplementary file 1 [file DataSheet1.docx]
